# Supramolecular DNA Three-Way Junction Motifs With a Bridging Metal Center

**DOI:** 10.3389/fchem.2019.00925

**Published:** 2020-01-15

**Authors:** Yusuke Takezawa, Mitsuhiko Shionoya

**Affiliations:** Department of Chemistry, Graduate School of Science, The University of Tokyo, Tokyo, Japan

**Keywords:** DNA, metal complex, artificial DNA, structural conversion, supramolecular chemistry, DNA nanotechnology

## Abstract

Various nano-sized supramolecular architectures have been constructed from DNA molecules via sequence-dependent self-assembly. A DNA three-way junction (3WJ), consisting of three oligonucleotides that are partially complementary to each other, is one of the simplest DNA supramolecular structures. This minireview covers studies on DNA 3WJ motifs bridged by an interstrand metal complex with some related works. The incorporation of interstrand metal complexes into DNA has attracted increasing attention because it potentially allows for metal-dependent regulation of the thermal stability and the structure of DNA supramolecules. Metal-bridged DNA 3WJs were synthesized from three DNA strands containing a bipyridine (bpy)-modified nucleotide in the presence of appropriate metal ions. The bpy-modified DNA strands were crosslinked by an interstrand 3:1 metal complex [Ni^II^(bpy)_3_ etc.] at the junction core. As a result, the thermal stability of the 3WJs was significantly enhanced upon metal complexation. Furthermore, metal-mediated structural transformation between DNA duplexes and 3WJs was demonstrated by using the same bpy-modified DNA strands. A mixture of bpy-modified strands and their natural complementary strands were self-assembled exclusively into duplexes in the absence of any transition metal ions. In contrast, addition of Ni^II^ ions induced the formation of 3WJs through the formation of an interstrand Ni^II^(bpy)_3_ complex, which served as a template for the 3WJ assembly. Because DNA 3WJ structures are essential structural motifs for DNA-based nanoarchitectures, the metal-mediated stabilization and structural induction of metal-locked 3WJs would lead to many potential applications to artificial DNA architectures.

## Introduction

A large variety of nano-sized supramolecular architectures have been constructed from DNA molecules via self-assembly, which can be precisely programmed by deliberate sequence design (Stulz and Clever, 2015). The sequence-dependent DNA self-assembly results in not only naturally-occurring duplex structures but also artificial supramolecular architectures including junctions, polyhedra, and DNA origami structures (Seeman, 2016). A DNA three-way junction (3WJ), consisting of three oligonucleotides that are partially complementary to each other, is one of the simplest DNA supramolecular structures. As three double helices are emanating from its branching point, 3WJ motifs serve as nodes or vertices of 2D and 3D DNA architectures. While the assembly of linear DNA duplexes yields only one-dimensional structures, the assembly of 3WJ motifs offers a versatile means to construct two-dimensional lattices, three-dimensional networks, polyhedra, dendrimers, and diverse intricate architectures.

In this minireview, studies on DNA 3WJ motifs bridged by an interstrand metal complex are thoroughly overviewed. The incorporation of interstrand metal complexes into DNA has attracted growing attention because it potentially allows for metal-dependent regulation of the thermal stability and the structure of DNA supramolecules. The most studied approach is the replacement of natural hydrogen-bonded base pairs in DNA duplexes by artificial metal-mediated base pairs, which are formed through coordination bonding between two ligand-type nucleosides and a bridging metal ion (Takezawa and Shionoya, 2012; Takezawa et al., 2017a,b; Müller, 2019). As metal coordination bonds are stronger than hydrogen bonds in general, incorporation of metal-mediated base pairs most often results in significant duplex stabilization (Tanaka et al., 2002a). Multiple incorporation of metal-mediated base pairs provides discrete metal arrays along DNA helices, exhibiting characteristic physical properties (Tanaka et al., 2003, 2006; Takezawa and Shionoya, 2014). Some metallo-base pairs are also used to induce conformational changes of DNA structures such as duplex–hairpin transformation (Kuklenyik and Marzilli, 1996; Böhme et al., 2007; Johannsen et al., 2010). Furthermore, a hydroxypyridone-based Cu^II^-mediated base pair is applied for the switching of the electrical conductivity of DNA devices (Liu et al., 2011) and of the catalytic activity of DNAzyme (Takezawa et al., 2019). The concept of interstrand metal complexation has been also applied to other higher-order DNA structures (Takezawa et al., 2015; Naskar et al., 2019), such as triple helices (Tanaka et al., 2002b; Takezawa et al., 2009) and G-quadruplex structures (Engelhard et al., 2013). Recently, DNA 3WJ structures bridged by an interstrand metal complex have been constructed in a manner analogous to the metal-bridged DNA helices mentioned above (Duprey et al., 2013; Stubinitzky et al., 2014; Takezawa et al., 2016). In addition to metal-dependent thermal stabilization of 3WJs, metal-mediated structural transformation between DNA duplexes and 3WJs was demonstrated. Since DNA 3WJ structures are essential structural motifs for DNA-based nanoarchitectures, the metal-mediated stabilization and structural induction of 3WJ motifs have many potential applications to artificial DNA architectures.

## Stabilization of DNA Three-Way Junction Motifs

**Figure 1:**
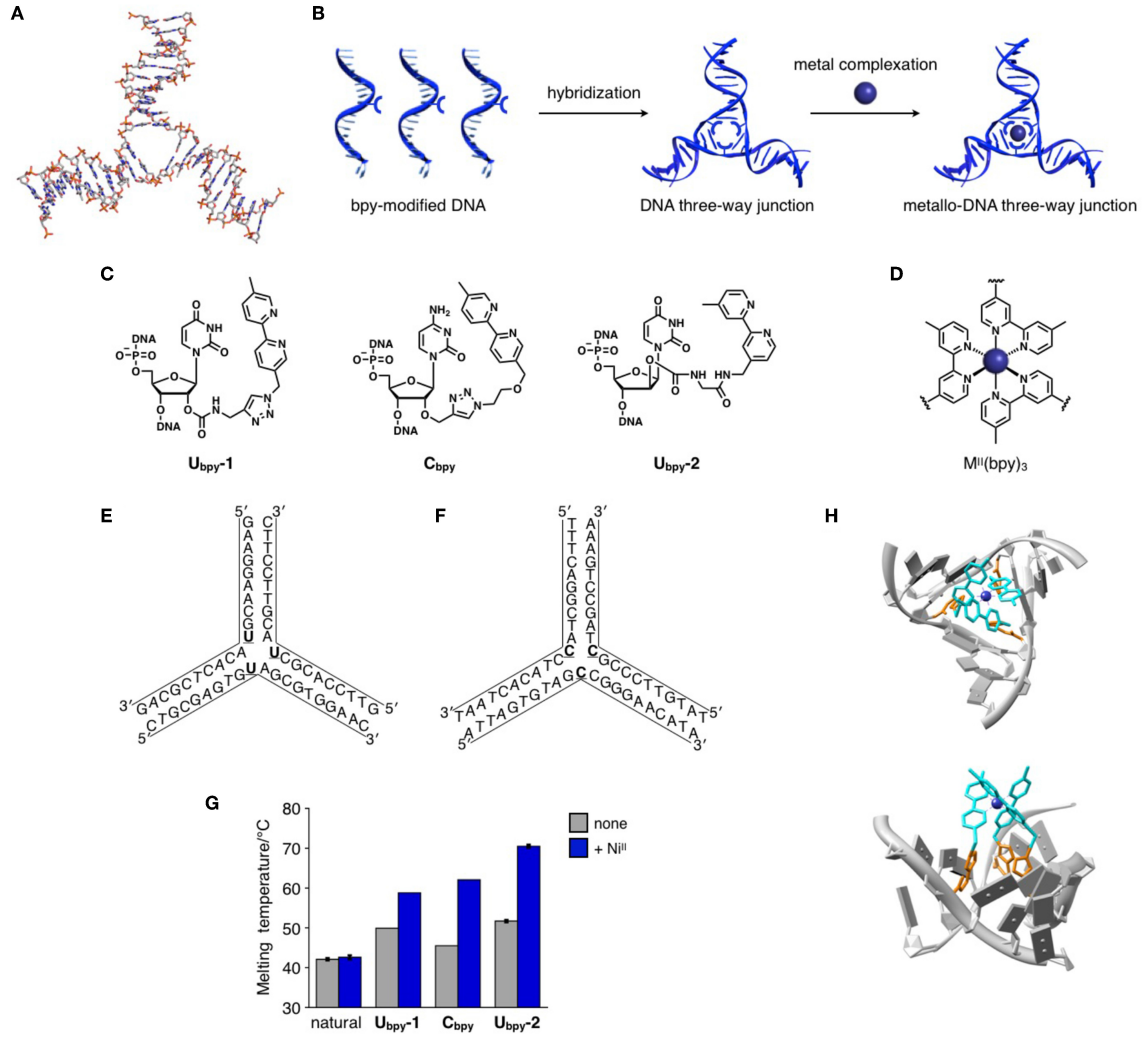
**(A)** An ideal structure of an unmodified DNA three-way junction (3WJ) motif. Drawn based on a crystal structure (PDB ID: 1DRG) reported by Baldwin et al. **(B)** Construction of a DNA three-way junction motif bridged by a metal complex. **(C)** Molecular design of bipyridine (bpy)-modified nucleotides. **(D)** The structure of a representative metal complex formed at the core of the 3WJ. **(E,F)** Base sequences of DNA strands forming metal-bridged 3WJs. U represents **U**_**bpy**_**-1** or **U**_**bpy**_**-2**. C represents **C**_**bpy**_. **(G)** Melting temperatures of the 3WJs in the absence and in the presence of Ni^II^ ions. [Ni^II^]/[3WJ] = 1.1 (for **C**_**bpy**_) or 1.0 (for the others). Note that the measurement conditions were slightly different form each other. For the details, see the original papers (Duprey et al., 2013; Stubinitzky et al., 2014; Takezawa et al., 2016). **(H)** Proposed structure of the Ni^II^(bpy)_3_ complex at the core of the **U**_**bpy**_**-1**-containing 3WJ. Only Λ-isomer is shown. Reproduced from a literature (Duprey et al., 2013) with permission from Wiley-VCH.

Figure 1A shows a typical structure of DNA 3WJs, which was revealed by X-ray analysis (Woods et al., 2001). A DNA 3WJ has a trigonal hydrophobic cavity at the center, which can be modified either covalently or non-covalently. Recent researches also focus on the development of molecules that bind to a central cavity of 3WJ through non-covalent interactions. Hannon et al. reported that a unique supramolecular metallo-helicate, [${\text{Fe}}_{2}^{\text{II}}$L_3_]^4+^, binds to a 3WJ cavity, and revealed its binding structure by X-ray structural analysis (Oleksi et al., 2006). The size and shape of the helicate fit well with the 3WJ cavity. Intermolecular interactions between the helicate and the 3WJ, electrostatic interactions and π-stacking, synergistically contributed to the binding. Vázquez and Vázquez López synthesized a chiral peptide helicate having two tris(bipyridine)–Fe^II^ complexes, which was conjugated to a foldon protein afterward (Gamba et al., 2014; Gómez-González et al., 2018). The ΛΛ-isomer of the helical metallopeptide was found to bind to a DNA 3WJ more strongly than the enantiomeric ΔΔ-isomer. Chenoweth et al. have synthesized triptycene-based 3WJ binders bearing positively charged side chains (Barros and Chenoweth, 2014). The binding of triptycene derivatives resulted in the significant enhancement of the thermal stability of 3WJs. More recently, a cationic azacryptand (Novotna et al., 2015) and a fluorescent calix[3]carbazole (Yang et al., 2018) have been developed as 3WJ binding compounds. A tetrahedral supramolecular metallo-cage, [${\text{Fe}}_{4}^{\text{II}}$L_4_]^8+^, was also reported to bind to 3WJs as well as mismatched DNA duplexes (Zhu et al., 2019). These 3WJ binders would be potential drug candidates because target 3WJ structures are found in the DNA replication fork as well as RNA secondary structures (Ducani et al., 2010; Barros et al., 2016). In the context of supramolecular nucleic acid chemistry, the 3WJ binding molecules are of great interest due to their future application as chemical inputs to stabilize or induce 3WJ-based supramolecular DNA architectures.

Covalent chemical modification is a promising way to functionalize 3WJ structures. Through covalent incorporation of functional units, the central cavity of 3WJs were utilized as a scaffold for chromophore assembly (Probst et al., 2012) and as a space for reactions (Hansen et al., 2009). Chemical modification also led to the thermal stabilization of 3WJs. Incorporation of pyrene-modified nucleotides into one of the three strands increased the stability of 3WJs (Filichev and Pedersen, 2003; Kumar et al., 2012). The 3WJ stabilization was also achieved by introducing a double-headed nucleoside having an additional nucleobase at the 2′-position (Jørgensen et al., 2011). These stabilization effects can be explained by the additional stacking interaction with the base pairs facing the central cavity. Stabilization based on the hydrophobic effect was also demonstrated by the incorporation of lipophilic spacers or side chains into three strands forming 3WJs (Laing and Juliano, 2015).

Interstrand metal complexation is another efficient strategy to stabilize 3WJ structures. A metal ligand can be incorporated into each strand so that the addition of appropriate metal ions leads to interstrand 3:1 ligand–metal complexation. As the ligand-modified 3WJs are stabilized only in the presence of appropriate metal ions, the thermal stability of the 3WJ can be tuned in a metal-responsive manner. Thus, the metal-dependent 3WJ stabilization is more advantageous in terms of applicability in supramolecular DNA chemistry. The design and properties of metal-bridged DNA 3WJs will be described in the following sections.

## Metal-Dependent Stabilization of Modified DNA Three-Way Junctions

The basic concept of the construction of metal-bridged DNA three-way junction (3WJ) structures is depicted in Figure 1B. Each DNA strand constituting a 3WJ motif can be modified with a metal-ligand, which forms a 3:1 ligand–metal complex at the branching point. As the three strands are additionally bridged by metal coordination bonds, the resulting metallo-DNA 3WJ was expected to be thermally stabilized. As a proof-of-concept example, we have chosen a bidentate bipyridine ligand (bpy), which is known to form stable complexes with various transition metal ions, for the construction of 3WJ structures bridged by an interstrand tris(bipyridine) metal complex. The bpy ligand was attached to the 2′-position of the ribose moiety because the reported crystal structures of natural 3WJs showed that the 2′-hydrogen atoms are directed to the center of the junction (Woods et al., 2001; Oleksi et al., 2006). Thus, the chemical modification at the 2′-position was thought to be most appropriate for the interstrand metal complexation at the junction core.

The structures of designed bpy-modified nucleosides and a representative tris(bipyridine) metal complex are shown in Figures 1C,D, respectively. We firstly designed **U**_**bpy**_**-1**, in which a bpy ligand was post-synthetically introduced via azide–alkyne Huisgen cycloaddition (Duprey et al., 2013). An analogous nucleoside **C**_**bpy**_, derivatized from 2′-propargyl cytidine, was reported by Wagenknecht (Stubinitzky et al., 2014). We also synthesized an improved version of bpy-modified nucleosides, **U**_**bpy**_**-2**, which has a bpy ligand at the 2′-position but on the opposite side (i.e., 2′-α position) through a carbamate linkage (Takezawa et al., 2016). These nucleosides were introduced in the middle of the DNA strands. When annealed, three bpy-modified strands self-assembled into 3WJ structures in which three bpy ligands are pre-organized in the junction core (Figures 1E,F). The formation of the 3WJ structures was confirmed by native polyacrylamide gel electrophoresis (PAGE) analysis (Takezawa et al., 2016). The metal complexation of M(bpy)_3_ complexes (M = Ni^II^ and Fe^III^) was confirmed based on UV spectroscopic analysis, showing a characteristic π-π^*^ transition absorption (Duprey et al., 2013; Stubinitzky et al., 2014). Electrospray ionization (ESI) mass spectrometry also provided evidence for the 1:1 binding of a metal ion and a bpy-modified 3WJ (Takezawa et al., 2016). All these analyses proved that each desired metal-bridged DNA 3WJ was formed with an appropriate transition metal ion.

UV-melting analysis clearly showed that the thermal stability of the bpy-modified 3WJs were significantly enhanced upon addition of metal ions (Figure 1G). In the presence of one equivalent of Ni^II^ ions, the melting temperature (*T*_m_) of a 3WJ possessing **U**_**bpy**_**-1** nucleosides (Figure 1E, U = **U**_**bpy**_**-1**) was increased from 49.9°C to 58.8°C (Δ*T*_m_ = +8.9°C) (Duprey et al., 2013). Titration of Ni^II^ ions showed that the highest stability was reached at a ratio [Ni^II^]:[3WJ] = 1:1. In contrast, Ni^II^ addition did not stabilize a natural 3WJ possessing T nucleosides instead of **U**_**bpy**_**-1** (Figure 1E, U = **T**) at all. Consequently, the Ni^II^-dependent stabilization was ascribed to the formation of an interstrand Ni^II^(bpy)_3_ complex at the junction core.

In the above-mentioned structure, **U**_**bpy**_**-1** nucleosides formally replaced thymidines (Ts) in a full-match 3WJ. On the other hand, when unpaired **C**_**bpy**_ nucleosides were additionally inserted into the branching point of the 3WJ (Figure 1F, C = **C**_**bpy**_), the 3WJ was stabilized by as much as +16.6°C upon addition of 1.1 equivalent of Ni^II^ ions (Stubinitzky et al., 2014). This larger stabilization compared with the case with **U**_**bpy**_**-1** could be attributed to its more flexible 3WJ scaffold.

A bpy-modified nucleoside more recently we reported, **U**_**bpy**_**-2**, further achieved increased metal-dependent 3WJ stabilization (Takezawa et al., 2016). A 3WJ containing three **U**_**bpy**_**-2** (Figure 1D, U = **U**_**bpy**_**-2**) showed sharper sigmoidal melting curves. While the 3WJ melted at 51.7°C under a metal-free condition, the melting temperature was drastically increased up to 70.5°C in the presence of Ni^II^ ions (Δ*T*_m_ = +18.8°C). The 3WJ stabilization observed here ranks as particularly large one even compared to the duplex stabilization caused by metal-mediated base paring (Takezawa et al., 2017a). It is worth to note that 3WJs containing only one or two bpy ligands showed no or less stabilization effects by Ni^II^ addition (Δ*T*_m_ = +0.7°C and +5.8°C, respectively). This result proved that all the three bpy ligands were involved in the metal complexation to form an interstrand Ni^II^(bpy)_3_ complex which provides the highest *T*_m_.

A modeling structure of the metal-bridged DNA 3WJ consisting of **U**_**bpy**_**-1** nucleosides is shown in Figure 1H. Due to the longer linker between the bpy ligand and the ribose moiety, the Ni^II^(bpy)_3_ complex resided just above the cavity. Thus, there is room for improving the degree of the stabilization through further optimization by linker design as well as by the use of other metal ligands.

Some of other divalent transition metal ions were found to stabilize the bpy-modified 3WJs. We have reported that the **U**_**bpy**_**-1**-modified 3WJ was stabilized by addition of equimolar amount of Fe^II^ (Δ*T*_m_ = +5.0°C) or Co^II^ ions (+3.3°C) (Duprey et al., 2013). In a similar fashion, the **C**_**bpy**_-modified 3WJ was stabilized by addition of Fe^III^ ions (Δ*T*_m_ = +16.7°C with 6 equiv. Fe^III^) or Zn^II^ ions (Δ*T*_m_ = +6.4°C with 1.1 equiv. Zn^II^) (Stubinitzky et al., 2014). The degree of the metal-dependent stabilization is reflected by the overall stability constants (β_3_) of the M^II^(bpy)_3_ complexes in aqueous media. For instance, the metal addition increased the melting temperatures of the **U**_**bpy**_**-1**-modified 3WJ in the order Ni^II^ > Fe^II^ > Co^II^, which agrees with the order of the β_3_ values reported for a simple bipyridine ligand (log β_3_ = 20.2, 17.2, and 15.9 for Ni^II^, Fe^II^, and Co^II^, respectively) (Smith and Martell, 1975). This result indicates that the thermal stability of the metal-bridged DNA 3WJ structures would be modulated according to the standard coordination thermodynamics.

Metal-bridged DNA 3WJs would be utilized as building blocks of higher-order DNA structures. Wagenknecht et al. attached perylene diimides (PDI) molecules at the termini of **C**_**bpy**_-modified 3WJs and investigated further DNA self-assembly (Stubinitzky et al., 2014). Thus, the strategy of metal-dependent 3WJ stabilization will be applied to metal-responsive regulation of the stability of DNA supramolecules and to the construction of stable DNA materials.

## Metal-Triggered Structural Conversion of Modified DNA Three-Way Junctions

**Figure 2:**
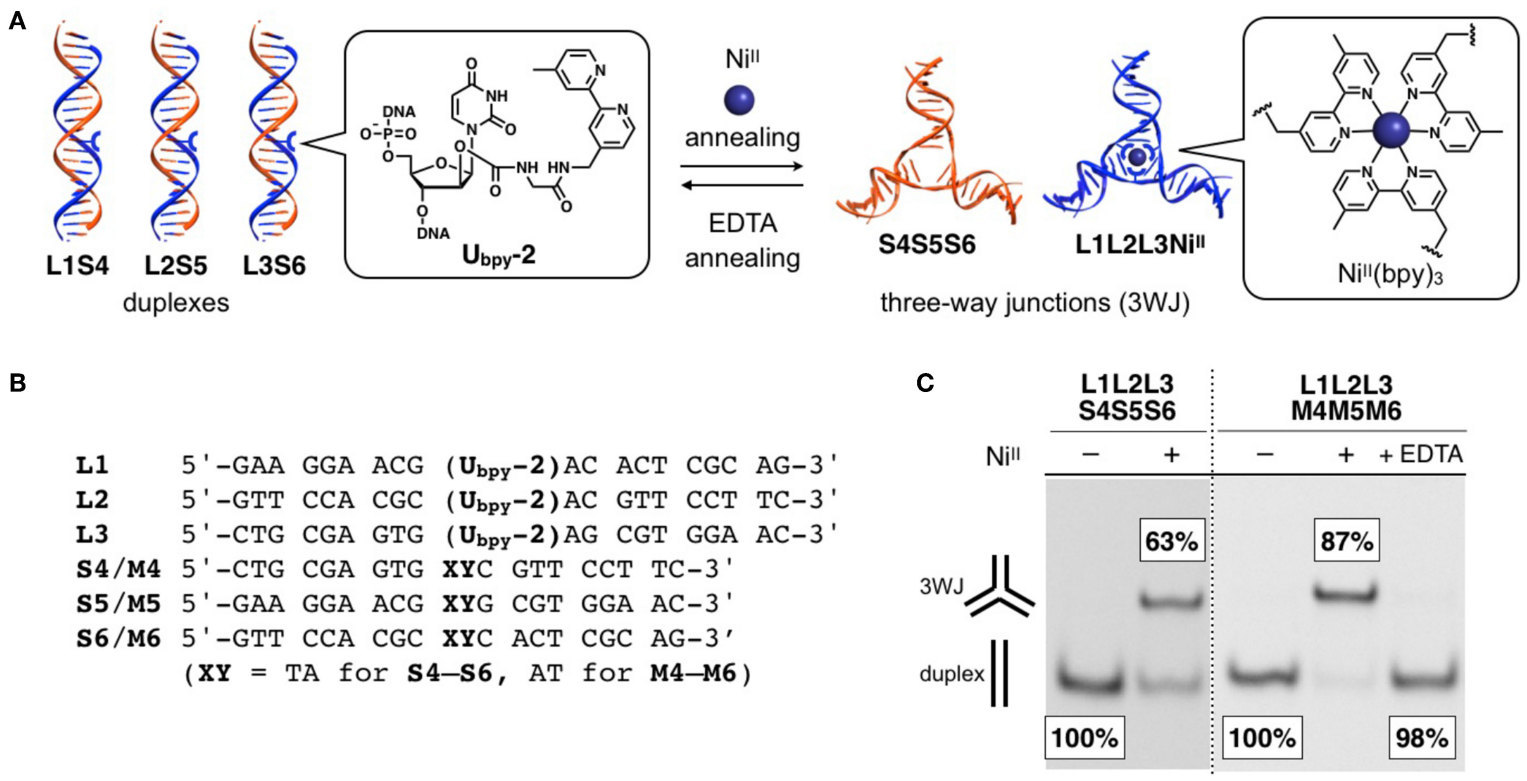
**(A)** Schematic representation of metal-triggered structural transformation between DNA duplexes and three-way junctions (3WJs). **(B)** Sequences of the DNA strands used for the metal-triggered structural conversion. **(C)** Native polyacrylamide gel electrophoresis (PAGE) analysis of the mixture of DNA strands in the absence and in the presence of one equivalent of Ni^II^ ions. “+EDTA” indicates the result after the removal of Ni^II^ ions by EDTA. Adapted from a literature (Takezawa et al., 2016) published by The Royal Society of Chemistry under the Creative Commons CCBY license.

Metal-triggered structural conversion has been a principal pillar of supramolecular chemistry. A number of stimuli-responsive supramolecules as well as molecular machines have been invented based on the dynamic metal–ligand coordination. This is also the case with supramolecular nucleic acid chemistry. Metal-mediated base pairs, especially those consisting of natural bases (i.e., T–Hg^II^-T and C–Ag^I^-C pairs), have been utilized for triggering structural changes in DNA duplexes, and further applied to the operation of DNA-based molecular switches and nanomachines (Liu et al., 2014; Wang et al., 2015; Lu et al., 2016). Metal-triggered structural conversion of DNA three-way junction (3WJ) motifs has been also of great interest in this field because 3WJs are pivotal components of DNA nanoarchitectures. The bipyridine (bpy)-modified DNA strands described above were utilized for Ni^II^-triggered duplex−3WJ conversion (Figure 2A) (Takezawa et al., 2016).

The system was comprised of three bpy-modified strands (**L1**, **L2**, and **L3**) and their complementary strands (**S4**, **S5**, and **S6**) (Figure 2B). Native PAGE analysis, in which one of the natural strands was labeled with a fluorophore for quantification, showed that the six DNA strands were self-assembled into three DNA duplexes (**L1S4**, **L2S5**, and **L3S6**) in the absence of transition metal ions. When one equivalent of Ni^II^ ions was added, two 3WJ structures, i.e., a metal-bridged 3WJ (**L1L2L3**·Ni^II^) and an unmodified 3WJ (**S4S5S6**), were formed in 63% yield (Figure 2C), showing Ni^II^-mediated transformation from duplexes to 3WJs. This structural conversion occurred as a result of the formation of an interstrand Ni^II^(bpy)_3_ complex, which served as a template for the 3WJ assembly. The maximum transformation efficiency was observed when 1.2 equiv. of Ni^II^ was added. This stoichiometry roughly corresponds to the 3:1 complexation of three bpy ligands and a Ni^II^ ion. Other transition metal ions such as Co^II^ also induced the 3WJ formation albeit in a substantially lower yield. Among late first-row transition metal ions, Ni^II^ showed the highest conversion efficiency. This result correlates with the largest overall stability constant (log β_3_ = 20.2), as is the case with the Ni^II^-dependent 3WJ stabilization discussed in the previous section.

More efficient duplex−3WJ transformation was demonstrated with mutated strands (**M4**, **M5**, and **M6** shown in Figure 2B). While these strands form a fully complementary 3WJ (**M4M5M6**), they form duplexes containing two mismatch pairs (**L1M4**, **L2M5**, and **L3M6**) with the bpy-modified strands. As a consequence, the thermal stabilities of the duplexes were lowered. The alteration in the relative stabilities resulted in the Ni^II^-mediated 3WJ formation in a sufficiently higher yield (Figure 2C). In addition, the subsequent removal of Ni^II^ ions by a chelating agent (EDTA) led to the quantitative regeneration of the duplexes. Accordingly, the metal-responsive reversible structural transformation was demonstrated between the duplexes and the 3WJs.

The efficiency of the metal-mediated 3WJ structural induction can be potentially improved by redesigning the interstrand metal complexes based on past achievements. Construction of 3WJ systems that are responsive to other types of metal ions may be also possible in theory. Thus, the metal-triggered 3WJ transformation would be a promising methodology to develop controllable DNA-based materials.

## Conclusions and Perspectives

DNA supramolecular architectures have been constructed conventionally based on the sequence-dependent hybridization. DNA self-assembly is programmable with the aid of theoretical prediction of the thermodynamic stability. The incorporation of interstrand metal complexes into DNA expands the scope of DNA-based supramolecular chemistry because metal coordination offers thermodynamic and kinetic characteristics different from hydrogen bonding-based DNA self-assembly. This minireview overviewed the development of DNA three-way junction (3WJ) structures bridged by an interstrand metal complex. Three types of bipyridine (bpy)-modified 3WJs have been reported and all of them were thermally stabilized by addition of transition metal ions such as Ni^II^. The metal-dependent stabilization was attributed to the formation of an interstrand complex (Ni^II^(bpy)_3_ etc.), which crosslinked the three oligonucleotides forming the 3WJ. Structural conversion between duplexes and 3WJs was further demonstrated in a Ni^II^-responsive manner. During the structural rearrangement, the formation of a Ni^II^(bpy)_3_ complex served as a trigger for the 3WJ assembly.

The design concept of the metal-bridged DNA 3WJs would have broad utility. The metal selectivity can be altered by selecting an appropriate metal ligand, and the efficiency of the stabilization and the structural conversion are in principle tunable based on the thermodynamics of the metal complex formation. Interstrand metal complexation may lead to the rigidification of the 3WJ structures, which will change the properties of 3WJ-based materials such as dendrimers and hydrogels (Yang et al., 2014; Wang et al., 2017). Chirality induction of interstrand metal complexes is also expected (Duprey et al., 2013). Furthermore, the strategy of metallo-DNA 3WJ formation with the use of 3:1 ligand–metal complexation would be employed for other types of DNA branched structures such as four-way junctions.

DNA junction structures are essential structural motifs for DNA-based nanoarchitectures. Therefore, the metal-mediated stabilization and structural induction of 3WJs would be applied to the development of metal-responsive DNA supramolecules and coordination-driven DNA molecular machines. Accordingly, the idea of the metal-bridged DNA 3WJs has many potential applications and provides a new insight in the field of supramolecular nucleic acid chemistry.

## Author Contributions

YT performed data analysis and prepared the manuscript. MS finalized the manuscript.

### Conflict of Interest

The authors declare that the research was conducted in the absence of any commercial or financial relationships that could be construed as a potential conflict of interest.
